# The sequelae of hematopoietic stem cell transplantation in adolescents and young adults: protocol for a systematic review

**DOI:** 10.1186/s13643-024-02560-x

**Published:** 2024-06-14

**Authors:** Nikita V. Baclig, Antonia Osuna-Garcia, Vivek Chotai, Patricia A. Ganz, Eden R. Brauer

**Affiliations:** 1 UCLA Fielding School of Public Health, David Geffen School of Medicine at UCLA, 10833 Le Conte Ave., CHS 60-054, Los Angeles, CA 90095 USA; 2 UCLA Louise M. Darling Biomedical Library, 12-077 Center for Health Sciences, Los Angeles, CA 90095-1798 USA; 3 University of California Los Angeles, Los Angeles, CA 90095 USA; 4 UCLA Fielding School of Public Health, David Geffen School of Medicine at UCLA, 650 Charles Young Drive South, Room A2-125 CHS, Los Angeles, CA 90095-6900 USA; 5 UCLA School of Nursing, 700 Tiverton Drive, Factor Bldg. Room 4-234, Los Angeles, CA 90095-6900 USA

**Keywords:** Adolescent and young adult, Hematopoietic stem cell transplantation, Survivorship

## Abstract

**Background:**

Hematopoietic stem cell transplantation (HSCT) is a life-saving treatment for adolescents and young adults (ages 15–39) with hematologic malignancy. Given the significant developmental milestones usually achieved during this unique life stage, this population is especially vulnerable to the interruption caused by a cancer diagnosis and its treatment. HSCT is a particularly invasive form of cancer therapy with many negative physical, social, and psychological sequelae. The long-term impact of HSCT in adolescents and young adults with hematologic malignancies warrants a systematic investigation of its effects to best shape clinical care and health policy.

**Methods:**

This protocol for a systematic review will focus on the long-term physical, psychological, social, spiritual, and health behavior effects experienced by adolescents and young adults who undergo HSCT for hematologic malignancy. We have constructed a specific search strategy that queries these five domains, which will be applied to five databases—Embase, PubMed, Cochrane Trials and Reviews, PsychInfo, and CINAHL—to identify the key literature. Two independent reviewers will perform a title/abstract screen followed by a full-text screen using standard screening templates to ensure the inclusion of outcomes in the post-acute HSCT period. Risk of bias will be assessed using the University of Adelaide Joanna Briggs Institute Collaboration Critical Appraisal Tools. Data from included studies will be abstracted on study characteristics, study setting, sample characteristics, and outcomes. Given the broad scope of the research question, data synthesis will focus on qualitative methods in accordance with Institute of Medicine standards.

**Discussion:**

While adolescents and young adults undergoing hematopoietic stem cell transplantation for hematologic malignancy are understood to have a unique survivorship experience, the sequelae of this treatment approach in this population have not been previously aggregated. This systematic review intends to expand insight into the adolescent and young adult experiences with HSCT in order to inform age-appropriate survivorship care and deliver this life-saving intervention with the best possible outcomes.

**Systematic review registration:**

PROSPERO CRD42022361663

## Background

Hematopoietic stem cell transplantation (HSCT) is a life-saving procedure for hematologic malignancies. However, it is associated with significant physical and mental hardship, causing a wide array of challenges in survivorship. Survivors frequently endorse fatigue and sleep disruption [1], psychiatric diagnoses such as depression and post-traumatic stress disorder (PTSD) [2, 3], fear of cancer recurrence [4], sexual dysfunction [5], and overall poor quality of life [6, 7].

Adolescent and young adult cancer patients are those diagnosed between age 15 and 39 years, according to the National Cancer Institute [8]. This group is particularly vulnerable to the life interruption that is caused by diagnosis and treatment. Having cancer as an AYA increases the risk of chronic physical conditions, subsequent cancers, and premature death [9]. Furthermore, AYAs with cancer often face disruptions in education and career trajectories as well as significant financial hardship [10, 11]. A systematic review from Barnett and colleagues summarized a wide array of issues for AYA cancer survivors including greater incidence of risky health behaviors, impaired fertility, body image distortion, psychosexual dysfunction, mental health symptoms, challenges to social well-being, and limited survivorship care [12].

While the most common AYA cancers—thyroid, breast, melanoma, and testicular—are not frequent indications for HSCT, the population of AYAs who are candidates for HSCT is considerable. New cases of Hodgkin lymphoma, leukemia, and non-Hodgkin lymphoma are diagnosed in 3.4, 3.6, and 4.0 per 100,000 individuals ages 15–39 years, respectively [13]. Reports from the Center for International Blood and Marrow Transplant Research (CIBMTR) suggest that the number of AYAs who undergo HSCT is growing, with nearly 3000 transplants in 2019, and that the most common indication for HSCT in AYAs remains hematologic malignancy, specifically acute myeloid leukemia (AML), Hodgkin lymphoma, and acute lymphoblastic leukemia (ALL) [14]. Furthermore, while the 5-year survival rates for AYAs with these malignancies are improving, they continue to be lower than those for children (ages 0–14), who generally have the best outcomes [15]. Among those who die in the post-acute transplant period (> 100 days), primary causes are generally not directly disease-related [14]. Thus, the population of AYA HSCT survivors is growing, yet they remain at risk for poor clinical outcomes in the survivorship period driven by non-cancer complications of their care. There remains significant equipoise in the literature about whether this risk is driven by physical, psychosocial, health behavior, or some combination of these factors.

The regular successful use of HSCT, arguably the most intensive cancer treatment available, to treat hematologic malignancies in the AYA population has resulted in a population of young cancer survivors with unique and significant sequelae. A rapid rise in the number of publications addressing these effects has increased awareness of the challenges this group faces, yet these findings have not been systematically indexed. Reviews from Mehta [16], Tewari [17], and others have initiated this investigation; however, none has addressed more recent findings in the literature, and all retain a focus on the physical consequences of HSCT in AYAs. Given that AYAs who undergo HSCT experience life interruption at a crucial moment in development, the psychosocial ramifications are noteworthy [18–20]. Here, we present a protocol for a systematic review of the literature addressing both the physical and non-physical sequelae of HSCT among AYA patients with hematologic malignancy.

**Fig. 1:**
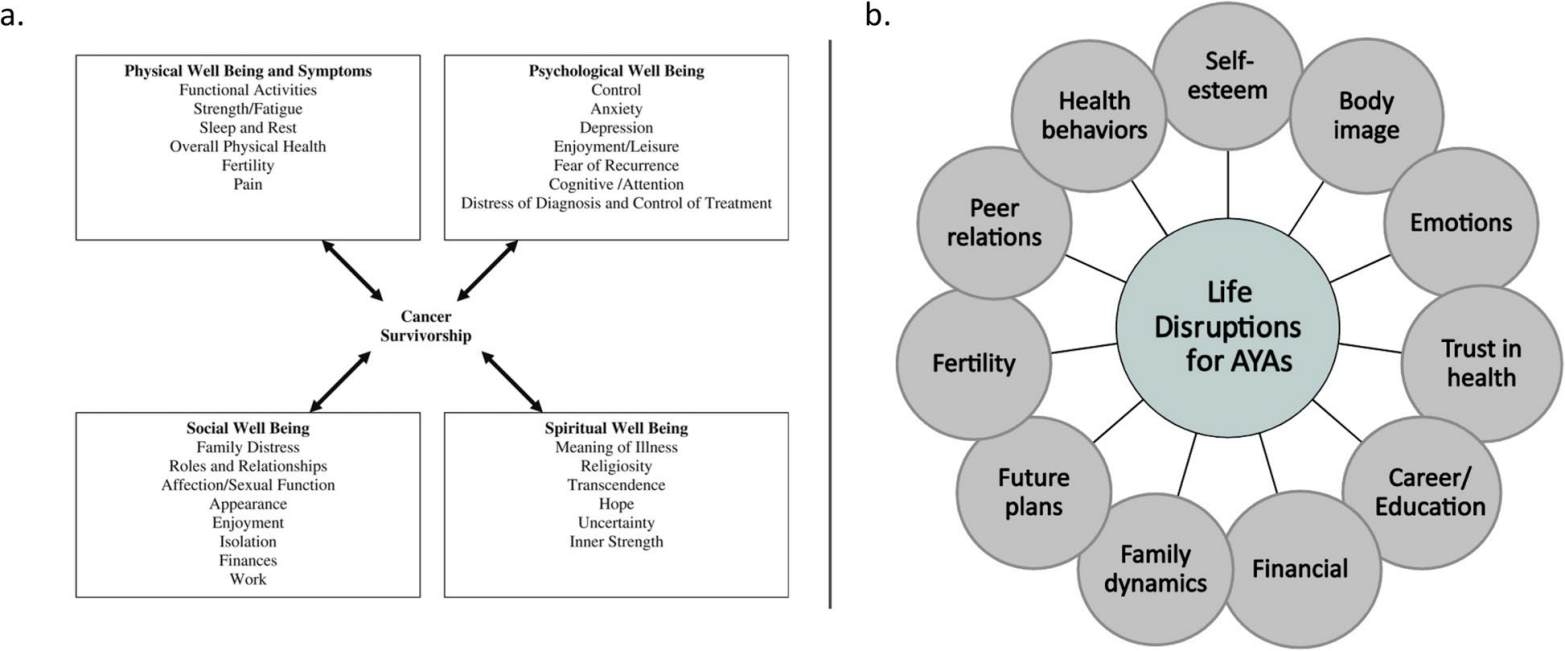
Established conceptual models. From these models, our conceptual framework (Fig. 2) was developed. **a** Quality of life: conceptual model, published in from Cancer Patient to Cancer Survivor:Lost in Transition [21]. **b** Life disruptions for adolescent and young adult (AYA) cancer patients, published in summary of the Institute of Medicine and Livestrong Foundation Workshop [22]

**Fig. 2:**
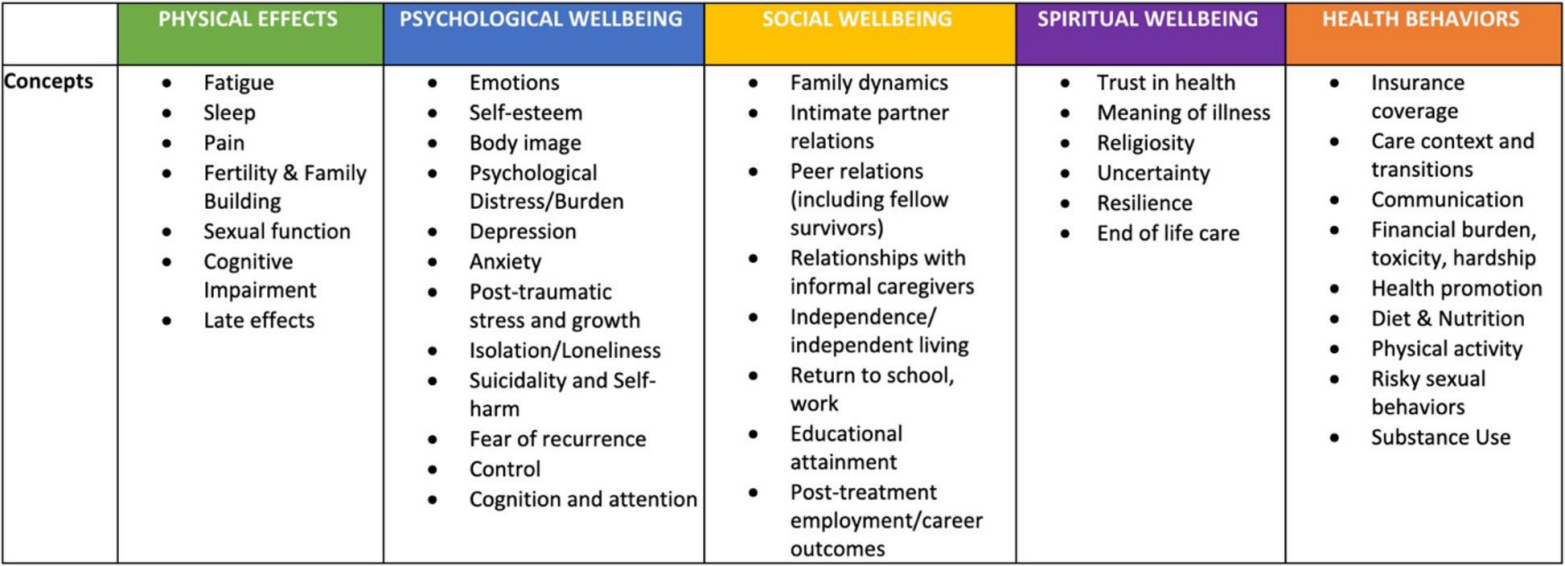
Conceptual Framework for Systematic Review. Captures the five main domains of anticipated sequelae of hematopoietic stem cell transplantation in adolescents and young adults with hematologic malignancies

This systematic review will address the long-term physical, psychological, social, spiritual, and health behavior effects of hematopoietic stem cell transplant for AYAs who undergo HSCT for hematologic malignancy. To define these concepts, we draw from models presented in two seminal texts: the Institute of Medicine’s “lost in transition” report [21] and an Institute of Medicine workshop addressing specific AYA patient needs [22]. Figure 1 shows the depictions of these original survivorship models, the first of which can be originally attributed to Ferrell and colleagues [23]. In this review, we coalesce these models into a single framework (Fig. 2) that conceptualizes the effects of HSCT among AYAs within five major domains: physical effects, psychological well-being, social well-being, spiritual well-being, and health behaviors. The primary aim of this review is to synthesize the known evidence describing the impact of HSCT among AYAs in order to identify avenues for further research and improved survivorship care in this unique population.

## Methods

This systematic review protocol was developed in accordance with the Preferred Reporting Items for Systematic Review and Meta-Analysis Protocols (PRISMA-P) guidelines [24]. The systematic review will include reports of original research published in English and indexed in online databases up to February 28, 2023. Unpublished manuscripts, conference abstracts, case studies, case-series reports, and articles without full-text manuscript availability will not be included. Studies will be drawn from a wide array of online databases to cover the literature from multiple scientific disciplines. Searches will be conducted in Embase, PubMed, Cochrane Trials and Reviews, PsychInfo, and CINAHL. Additional papers will be identified by hand review of the included articles’ bibliographies.

Our focus will be on patients who received a diagnosis of hematologic malignancy between the ages of 15–39 years and subsequently underwent HSCT. Survivors of malignancies treated in childhood or in older adulthood will be excluded. Studies will only be included if data is stratified by age at cancer diagnosis or if the study is limited to AYA patients. Similarly, we are interested in recipients of HSCT for hematologic malignancies, so AYA patients who received HSCT for other indications (i.e., for breast cancer, testicular cancer, or benign hematologic conditions) will be excluded. As our research question focuses on long-term survivorship and the risk of death from acute complications of HSCT is highest in the first 100 days, we will exclude studies that only describe the effects during the first 100 days post-transplant [25]. Studies will be included if the outcomes reported incorporate at least one of the conceptual domains described above. Notably, we will include both positive and negative outcomes of HSCT, as both fit within the defined domains.

**Fig. 3:**
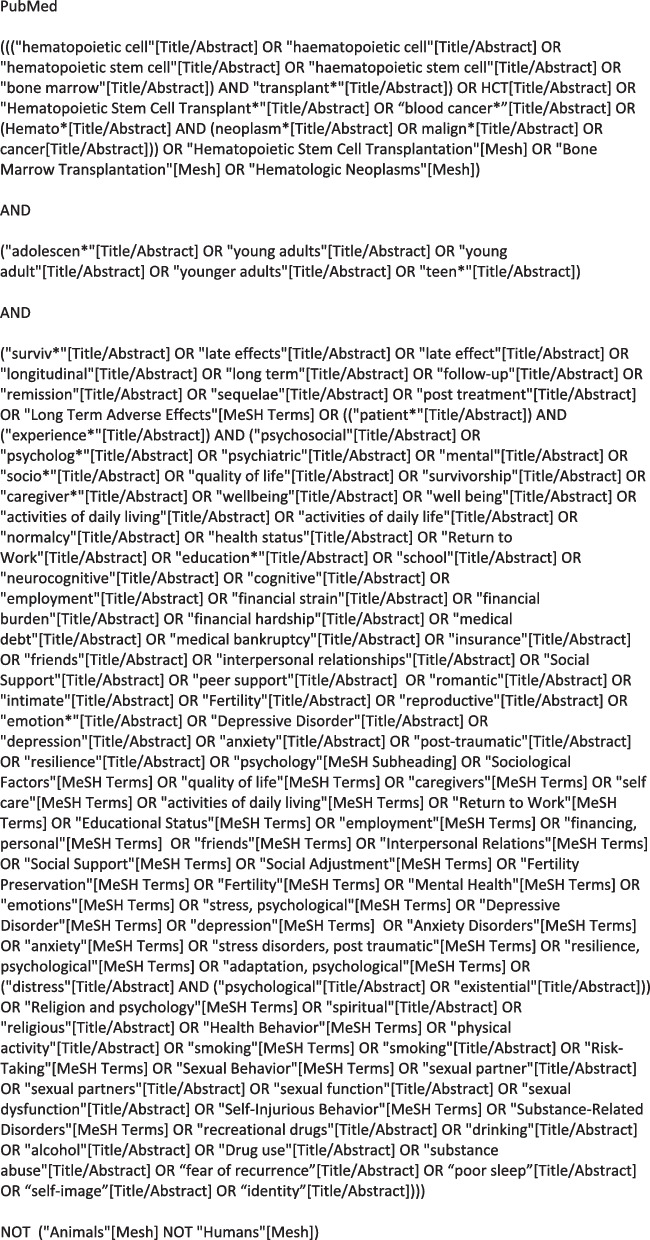
Search strategy. Example of literature search strategy for PubMed. Strategies for other databases were designed to reflect the content of the PubMed search

In collaboration with colleagues in the UCLA Louise M. Darling Biomedical Library, a finalized search strategy was developed based on the five survivorship domains and created to maximize both inclusivity and discernment around this unique research question. The final search strategy will encompass the concepts of “cancer” or “hematopoietic stem cell transplantation” and “adolescents and young adults” and “survivorship”. Please see Fig. 3 for an example of a complete search for PubMed. The search strategy will be translated as appropriate for all databases investigated.

**Fig. 4:**
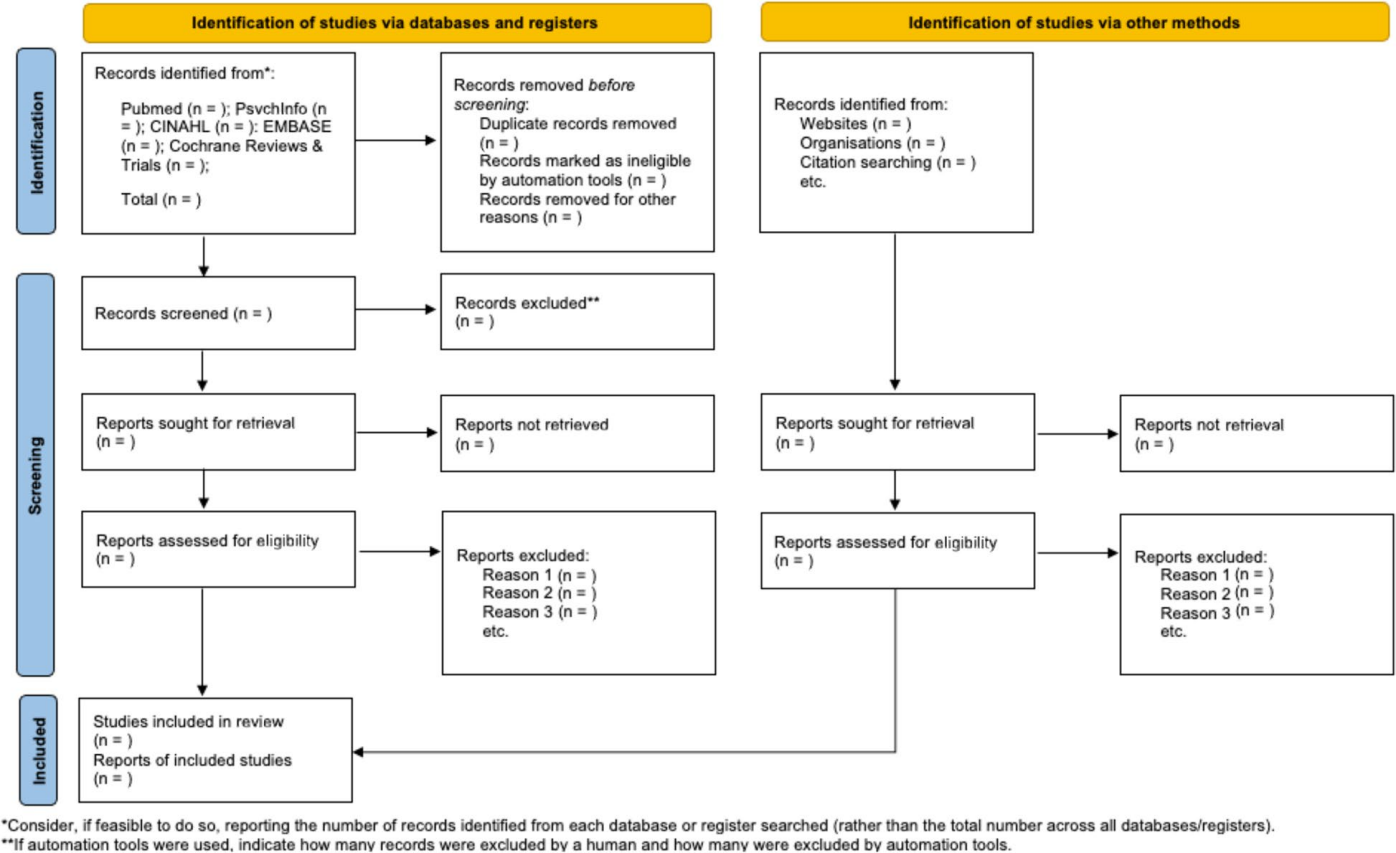
Proposed flow diagram. Derived from PRISMA 2020 flow diagram for new systematic reviews [27]

All search results will be managed using Rayyan Intelligent Systematic Review [26]. Search results from individual databases will be uploaded directly into Rayyan, and duplicates will be removed before screening. All screening decisions will be recorded according to the Preferred Reporting Items for Systematic Reviews and Meta-Analyses (PRISMA) guidelines (see Fig. 4). Screening of search results will commence with initial title/abstract screening by two independent, blinded reviewers. Regular reviewer meetings will be held, at first to identify points of clarification in screening protocols and then to discuss and settle any conflicting screening decisions. Inter-rater reliability will be calculated from the majority of screened articles, once the screening protocol has been tested and established. Any disagreements that cannot be resolved will be evaluated by a third reviewer. The decision rationale for each article reviewed will be documented.

Articles that fail to be excluded by pre-determined criteria at this stage or articles for which more information is needed will move to a second, full-text screening stage. In the full-text stage, complete manuscripts will be attached to Rayyan entries for more in-depth review. Once again, two independent, blinded reviewers will conduct full-text screening according to established criteria. Inter-rater reliability will be calculated for the totality of full-text reviews. The decision rationale for each publication will again be documented. With regular meetings, we will attempt to reach a consensus should disagreement about inclusion arise. However, should consensus not be achieved, a third blinded reviewer will be incorporated.

Critical appraisal will then be assessed for each study included after full-text screening. University of Adelaide JBI Collaboration Critical Appraisal Tools will be used for critical appraisal and risk of bias assessment [28]. JBI tools were selected over others (such as ROBINS-I or CASP) primarily because we anticipate studies addressing this topic to be widely varied in study design and the JBI tools offer checklists tailored for different study structures. For each study, we will identify which checklist most appropriately reflects the design of the study and both reviewers will independently evaluate. Inter-rater reliability will be calculated. Studies will be included if > 70% of responses to checklist criteria are “yes”. If the two reviewers’ assessments are discordant, a third reviewer will assess. Should any study not have an appropriate checklist for risk of bias assessment using the JBI tools, it will be included to encompass as much of the literature as possible. Such articles will be marked as having not been assessed for bias.

Once articles for inclusion are finalized, data extraction will be performed independently by two reviewers. A detailed abstraction guide will be drafted, piloted, and adapted if needed to ensure standardized responses from all reviewers. Data will then be abstracted directly into a structured evidence table, with abstracted data blinded to other reviewers until the time of the joint review. Abstracted data will be jointly reviewed at regular intervals, and any discrepancies will be discussed until a resolution is reached. Data variables and outcomes to be abstracted are listed in Table 1. Given the nature of this review, outcomes will vary by article but will address at least one of the concepts included in the five domains of our conceptual framework (see Fig. 2). There will be no prioritization of outcomes as articles addressing any concept will be included in our qualitative review. Both first-order constructs (rates, distributions, proportions, direct study subject quotes) as well as second-order constructs (author interpretations, conclusions, or ideas) will be included as study outcomes.

**Table 1:** Data variables for extraction and their definitions

**Category**	**Variable**	**Definition**
Publication specifications	Author(s)	First and last names of all authors
	Date	Date of initial publication
	Publication ID	PMID, doi, and/or ISSN
	Journal	Name of journal where originally published
	Database	Database source(s) from which the article was identified
Study characteristics	Design	Study design as defined by Campbell and Stanley (citation)
	Aim	Objective of the study or primary research question it attempts to answer
	Intervention	Exposure or treatment whose effect is being studied
	Comparison	Defined group to whom the intervention is being compared, if applicable
	Inclusion criteria	Subject characteristics necessary to be included in the study
	Exclusion criteria	Subject characteristics that prevented inclusion in the study
	Data source	Type of data that was collected and analyzed in the study (i.e., medical records, insurance databases, survey responses)
	Study instruments	List any standardized scientific instruments used (i.e., PHQ-9 to measure depression)
	Analysis	Planned statistical analysis described by study authors
Study setting	Location	Physical context for the study including facility (i.e., hospital vs. clinic) and geographic location (city, state, country)
	Time	Period of time in which data was collected including start and end dates
	Population	Larger group from which study subjects selected
Sample characteristics	Size	Number of subjects included in the study
	Percentage AYA	Percentage of study subjects who were between 15 and 39 years old at the time of study inclusion
	Age at diagnosis	Average age of cancer diagnosis among study subjects
	Age at HSCT	Average age of HSCT among study subjects
	Male sex	Percentage of study subjects who were male
	Race/ethnicity	Distribution of races/ethnicities among study subjects
	Insurance	Distribution of insurance coverage status among study subjects
	Malignancy	Distribution of patients with leukemia, Hodgkin lymphoma, and non-Hodgkin lymphoma
	Conditioning regimen	Distribution of various conditioning regimens used for HSCT
Outcomes	Primary outcome	The main finding of the study
	Secondary outcomes	Additional study findings
	Domain addressed	Which of the following conceptual domains were addressed in study outcomes: physical, psychological, social, spiritual, and health behaviors
	Strengths	Author reported the strengths of the study
	Limitations	Author reported the limitations of the study

While data synthesis often involves both qualitative and quantitative analysis [29], we anticipate that the wide scope of this review will result in outcomes that cannot feasibly be quantitatively synthesized. Thus, we will focus primarily on qualitative synthesis. Data abstracted into evidence tables will be coalesced into summary tables by domain. In accordance with the Institute of Medicine standards for qualitative analysis [30], we will focus our synthesis on a descriptive review of the clinical and methodological characteristics of included studies, emphasizing strengths, limitations, and how bias could compromise the reported results. We will also identify patterns in findings across studies and examine how individual study characteristics impact these common themes and summative deductions. Meta-bias will be assessed across studies with attention to publication bias specifically. Finally, we will discuss the relevance of the included studies to our population of interest, identifying the gaps in the literature and opportunities for further study. Given the nature of the data, we will similarly forego formal assessment of confidence in cumulative data as tools such as the Grading of Recommendations Assessment, Development, and Evaluation (GRADE). Any major protocol amendments will be documented and resubmitted for review.

## Discussion

While AYA patients who received HSCT for hematologic malignancy are understood to have a unique survivorship experience, the sequelae of transplantation in this population have not been previously aggregated. As a result, there are limitations to further research and clinical innovation for this population. This systematic review intends to expand our insight into AYA experiences with HSCT to deliver this life-saving intervention with the best possible outcome.

## Data Availability

Not applicable.

## Abbreviations

AYAs: Adolescents and young adults
HSCT: Hematopoietic stem cell transplantation
PTSD: Post-traumatic stress disorder
UCLA: University of California in Los Angeles
